# Efficacy of baloxavir marboxil on household transmission of influenza infection

**DOI:** 10.1186/s40780-020-00178-4

**Published:** 2020-10-01

**Authors:** Takumi Umemura, Yoshikazu Mutoh, Takato Kawamura, Masayuki Saito, Takahito Mizuno, Aiko Ota, Koji Kozaki, Tetsuya Yamada, Yoshiaki Ikeda, Toshihiko Ichihara

**Affiliations:** 1 Department of Pharmacy, Tosei General Hospital, 160, Nishi oiwakecho, Seto, Aichi 489-8642 Japan; 2 Department of Infection and Prevention, Tosei General Hospital, 160, Nishi oiwakecho, Seto, Aichi 489-8642 Japan; 3 College of Pharmacy, Kinjo Gakuin University, 2-1723, Omori, Moriyama-ku, Nagoya, Aichi 463-8521 Japan

**Keywords:** Baloxavir marboxil, Oseltamivir phosphate, Secondary attack rate, Influenza

## Abstract

**Background:**

Baloxavir marboxil (baloxavir) is a new anti-influenza virus agent that is comparable to oseltamivir phosphate (oseltamivir). Since the efficacy of baloxavir in preventing household transmission of influenza is not well established, we compared the secondary household influenza virus transmission rates between patients on baloxavir vs oseltamivir.

**Methods:**

Between October 2018 and March 2019, we enrolled index patients (diagnosed with influenza and treated with baloxavir or oseltamivir) and household members. The secondary attack rate of household members was compared between index patients treated with baloxavir vs oseltamivir. Risk factors of household transmission were determined using multivariate logistic analyses.

**Results:**

In total, 169 index patients with influenza type A were enrolled. The median age was 27.0 (interquartile range; 11–57) years. The number of index patients treated with baloxavir and oseltamivir was 49 and 120, respectively. The secondary attack rate was 9.0% (95% confidence interval [CI]: 4.6–15.6) in the baloxavir group and 13.5% (95% CI: 9.8–17.9) in the oseltamivir group. In the multivariate analysis, independent risk factors were 0–6 years of age (odds ratio [OR] 2.78, 95% CI: 1.33–5.82, *p* < 0.01) and not being on baloxavir treatment. (OR: 0.63, 95% CI: 0.30–1.32, *p* = 0.22).

**Conclusion:**

The household secondary attack rate of influenza was comparable in patients treated with baloxavir vs oseltamivir. Therefore, baloxavir can be used as an alternative therapy to oseltamivir in reducing household transmission of influenza.

**Trial registration:**

Patients in this study were retrospectively registered. https://www.tosei.or.jp/clinical/pdf/2_influenza.pdf.

## Introduction

One of the major ways of transmitting the influenza virus is through household contact [1]. As the 2009 influenza A (H1N1) virus spread globally, many countries implemented mitigation policies that included home isolation of persons with confirmed H1N1 infection [1]. This increased the risk of infection to other household members. The risk factors for H1N1 transmission (to other household members) include the presence of young children in the household, household size, and use of antiviral agents, such as neuraminidase inhibitors [oseltamivir phosphate (oseltamivir), zanamivir hydrate (zanamivir), laninamivir octanoat (laninamivir), and peramivir hydrate (peramivir)] [2–6].

Early oseltamivir treatment is effective in reducing the duration of symptoms and the risk of household transmission [3, 5]. Furthermore, the use of zanamivir within 48 h of symptom onset reduced the risk of household transmission [6]. However, the efficacy of reducing household transmission of H1N1 varies among different neuraminidase inhibitors [7].

Baloxavir marboxil (baloxavir) is a new class of antiviral agent that works as a cap-dependent endonuclease inhibitor. Baloxavir has been shown to be superior to oseltamivir in reducing the viral load 1 day after initiation of the trial regimen in patients with uncomplicated influenza [8]. Therefore, we conducted a single-center observational study to test our hypothesis that baloxavir can be used as an alternative therapy to oseltamivir in preventing household transmission of influenza.

## Materials and methods

### Setting and population

This study was a retrospective, single-center study conducted at the emergency medical department in Tosei General Hospital, Japan.

Between October 2018 and March 2019, we enrolled index patients with confirmed influenza A who were treated within 48 h with either baloxavir or oseltamivir (confirmed by phone call). Similarly, family members of the confirmed cases were enrolled in the study. Influenza infection was diagnosed using rapid influenza diagnostic tests (RIDTs), Quick Chaser Flu A, B (Mizuho Medy), from the nasopharyngeal specimen. Family members who had previously undergone prophylactic treatment for influenza were excluded from the study.

Both the patient and the family members provided informed consent prior to being included in the study, and the study was approved by the Ethics Committee of Tosei General Hospital (receipt No. 797).

### Data collection

Clinical data were collected from medical records or by phone call. For index patients, we collected data on the age, sex, status of influenza vaccination in the same season, body temperature, and presence of respiratory tract symptoms when influenza was diagnosed. For household members, we collected data on the age groups, household size, influenza vaccination in the same season, presence of household transmission, and time duration from the illness onset of the index case. The age groups were stratified into the following four groups according to a past report and modified to the Japanese style: pre-school children (0–6 years), underage (7–19 years), adults (20–64 years), and elderly people (≥ 65 years) [4]. Furthermore, the household size were stratified into the following three groups according to a past report: ≤ 3, 4 and ≥ 5 [3].

### Variables

An index patient was defined as a patient diagnosed with influenza and on treatment with either baloxavir or oseltamivir. The dose of the test products was based on the package insert: Adults, baloxavir 40 mg (over 80 kg, 80 mg) single-dose administration or oseltamivir 75 mg twice daily for 5 days; Children, baloxavir 40 mg (over 40 kg) or 20 mg (20–40 kg) single-dose administration or oseltamivir 2 mg/kg twice daily for 5 days.

Family members included in the study were those who lived with the index case. Household transmission was defined as household members who were diagnosed with the same influenza type as the index patient between 1 and 7 days after the onset of symptoms in the index patient [6, 7].

### Statistical analysis

The primary outcome was the secondary attack rate in the baloxavir and oseltamivir groups. Secondary attack rate was determined as the proportion of household members who were infected. The 95% confidence interval (CI) of the secondary attack rates was estimated using the Clopper–Pearson method [9]. The effect of baloxavir on transmission was estimated using univariate and multivariate logistic regression analyses, including other factors. In patients’ backgrounds, qualitative and stratified continuous variables were compared using the Fisher exact or Pearson chi-square test. Continuous variables were compared using the Student t-test or the Mann–Whitney U test, as appropriate. Multivariate logistic analyses were used for the logistic regression models, and variables achieving a probability *p-*value of < 0.2 in the univariate logistic analysis were included in the multivariate analysis [10]. Predictive values were presented as odds ratios (ORs) with the respective 95% CI. Two-tailed *p* values < 0.05 were considered statistically significant, and analyses were performed using IBM SPSS Statistics ver. 25 (IBM®).

## Results

**Fig. 1:**
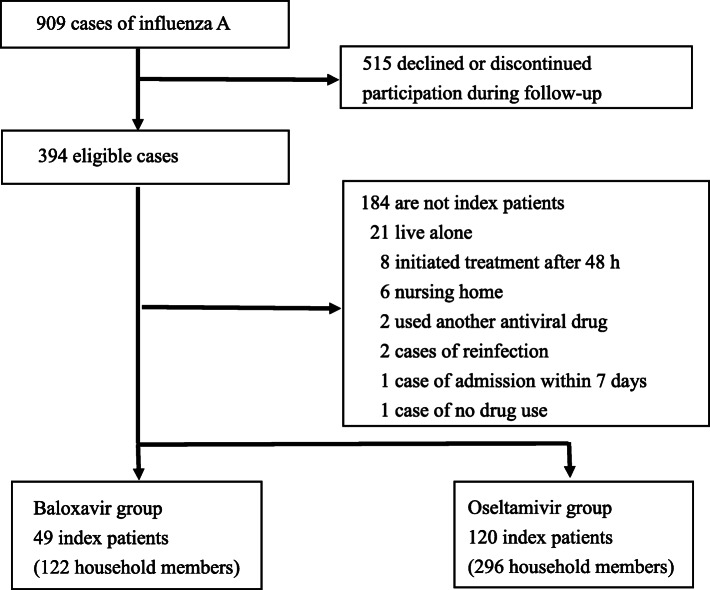
Flow chart of extraction of the index patients, treated with baloxavir and oseltamivir for influenza infection, and their household members

During the study period, 909 patients were confirmed with influenza A; of whom, 169 index patients (18.6%) were eligible for this study. The median age was 27.0 (interquartile range (interquartile range (IQR); 11–57) years, and 92 of the 169 patients were male (54.4%). Forty-nine index patients and 122 household members in the baloxavir group and 120 index patients and 296 household members in the oseltamivir group were eligible for this study. The exclusion criteria are shown in Fig. 1.

No significant difference was observed in the baseline characteristics of index patients who received baloxavir and oseltamivir and their household members in the two groups (Tables 1 and 2). Further, not all of the other variables were significant.

**Table 1:** Baseline characteristics of the index patients

Variables	Baloxavir group	Oseltamivir group	*P* value
N	49	120	
Age of index patients (years), median (IQR)	33 (13–53)	26 (10–56)	0.34
0–6	2 (4.1)	20 (16.7)	
7–19	14 (28.6)	27 (22.5)	
20–64	24 (49.0)	55 (45.8)	
≥ 65	9 (18.3)	18 (15.0)	
Male, n (%)	27 (55.1)	65 (54.2)	0.82
Influenza vaccination in the same season, n (%)	12 (24.5)	46 (38.3)	0.08
Body temperature (°C) mean ± SD	38.7 ± 0.94	38.4 ± 1.73	0.42
Respiratory tract symptom, n (%)	22 (44.9)	46 (38.3)	0.43

*IQR* Interquartile range, *SD* Standard deviation

**Table 2:** Baseline characteristics of the household members

Variables	Baloxavir group	Oseltamivir group	*P*-value
N	122	296	
Age of household members (years), n (%)			0.20
0–6	10 (8.2)	31 (10.5)	
7–19	27 (22.1)	47 (15.9)	
20–64	73 (59.8)	171 (57.7)	
≥ 65	12 (9.9)	47 (15.9)	
Household size			0.14
≤ 3	29 (23.8)	87 (27.4)	
4	55 (45.1)	108 (36.5)	
≥ 5	38 (31.1)	101 (34.1)	
Influenza vaccination in the same season, n (%)	30 (24.6)	99 (33.4)	0.08

Table 3 shows secondary attack rates of household members in individual models. The secondary attack rate in the baloxavir group (9.0%) was not statistically different from that in the oseltamivir group (13.5%) (*p* = 0.34 by Pearson chi-square test). When stratified by age, the secondary attack rate was highest in the 0–6-year age group (27.5%; 95% CI: 15.9–41.7).

**Table 3:** Secondary attack rates of household members in individual models

	n	Secondary attack rates	95% CI
Age of household patients (years)			
0–6	47	27.5	15.9–41.7
7–19	69	13.5	6.7–23.5
20–64	243	12.1	8.3–16.8
≥ 65	59	12.7	6.7–23.5
Age of index patients (years)			
0–6	67	18.3	10.1–29.3
7–19	128	12.1	7.1–18.9
20–64	167	14.5	9.6–20.7
≥ 65	56	13.6	6.0–25.0
Household size			
≤ 3	116	14.8	9.0–22.3
4	163	14.9	9.9–21.2
≥ 5	139	13.1	8.1–19.7
Influenza vaccination in the same season in household patients			
Yes	140	14.3	8.9–21.2
No	278	14.2	10.5–18.8
Index patients with treatment			
Baloxavir	122	9.0	4.6–15.6
Oseltamivir	296	13.5	9.8–17.9
Respiratory tract symptoms in index patients			
Yes	157	15.9	10.6–22.6
No	261	13.3	9.5–17.9

*CI* Confidence interval

**Fig. 2:**
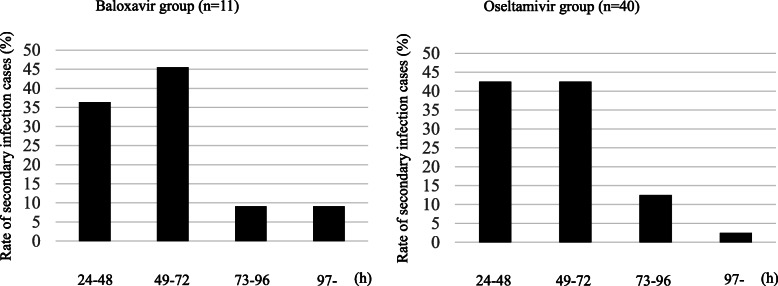
Time from illness-onset in the index case to illness-onset in secondary cases in the households of patients in the baloxavir and oseltamivir groups

Figure 2 shows the rate of secondary infections from illness onset in the index case to illness onset in secondary cases. The median duration was 2 days (IQR 2–4) for both groups. In the baloxavir group, the duration from illness onset in the index case to illness onset in the secondary case among 11 out of 15 cases was less than 3 days (73.3%). Similarly, in the oseltamivir group, the duration from illness onset in the index case to illness onset in the secondary case among 40 out of 48 cases was below 3 days (83.3%).

Table 4 shows the results of univariate analysis of factors affecting the household transmission of influenza virus. In the univariate analysis, variables with *p*-values < 0.2 included aged 0–6 years of household patients and index patient on baloxavir. The results of multivariate analysis are shown in Tables 5. No statistically significant differences were observed in index patients on baloxavir treatment and the aged 0–6 years was an independent risk factor (OR: 2.78, 95% CI: 1.33–5.82, *p* < 0.01).

**Table 4:** Univariate analyses of factors affecting household transmission of influenza infection

Variables	With household transmission (*n* = 51)	Without household transmission (*n* = 367)	Odds ratio (95% CI)	*P* value
Age of household patients (years), n (%)				
0–6	12 (23.5)	35 (9.5)	2.92 (1.40–6.09)	< 0.01
7–19	7 (13.7)	62 (16.9)	0.93 (0.42–2.08)	0.87
20–64	27 (52.9)	216 (58.8)	0.77 (0.42–1.38)	0.38
≥ 65	5 (9.8)	54 (14.7)	0.63 (0.24–1.66)	0.34
Age of index patients (years), n (%)				
0–6	7 (13.7)	60 (16.3)	0.81 (0.35–1.89)	0.63
7–19	14 (27.4)	114 (31.1)	0.84 (0.44–1.61)	0.60
20–64	23 (45.1)	144 (39.2)	1.29 (0.71–2.32)	0.40
≥ 65	7 (13.7)	49 (13.4)	1.03 (0.44–2.42)	0.94
Household size, n (%)				
≤ 3	15 (29.4)	101 (27.5)	1.10 (0.58–2.09)	0.78
4	22 (43.1)	141 (38.4)	1.22 (0.67–2.20)	0.52
≥ 5	14 (27.4)	125 (34.1)	0.73 (0.38–1.41)	0.35
Influenza vaccination in the same eason in household patients, n (%)	18 (35.3)	117 (31.9)	1.17 (0.63–2.16)	0.63
Index patients with baloxavir treatment, n (%)	10 (19.6)	108 (29.4)	0.59 (0.28–1.21)	0.14
Respiratory tract symptoms in index patients, n (%)	23 (45.1)	132 (36.0)	1.46 (0.81–2.64)	0.31

*CI* Confidence interval

**Table 5:** Multivariate analysis of factors affecting household transmission of influenza infection

Variables	Odds ratio (95% CI)	*P*-value
0–6 years of household patients	2.78 (1.33–5.82)	< 0.01
Index patients treated with baloxavir	0.63 (0.30–1.32)	0.22

*CI* Confidence interval

## Discussion

Our observational study indicates that there is no association between baloxavir and reduced household transmission. Baloxavir is a novel antiviral agent that is administered as a prodrug and must be hydrolyzed to its active form for anti-influenza activity. Baloxavir blocks influenza virus proliferation by inhibiting the initiation of mRNA synthesis [11]. This virucidal action results in viral load reduction 1 day after initiation of the trial regimen in patients with uncomplicated influenza [8].

Household transmission is one of the major ways of spreading the influenza virus. Early reduction of viral load may be necessary to avoid influenza transmission [12], and several reports have shown that anti-influenza drugs may reduce household transmission [3, 5]. To our knowledge, this is the first study to compare the efficacy of baloxavir to oseltamivir in reducing secondary influenza virus transmission. For the early prevention of viral shedding in the infection, it is important to initiate antiviral treatment within 48 h [13]. This study enrolled patients who had undergone antiviral treatment within 48 h. Both groups of patients (baloxavir and oseltamivir) had similar time-trends from illness-onset in the index case to illness-onset in secondary cases in the households (Fig. 2). Previous studies have shown that the longest duration from the onset of illness in index patients to illness onset in secondary cases in the households is 24–48 h with neuraminidase inhibitor treatment [6, 14]. Our results in the oseltamivir groups are similar to past reports.

Although baloxavir reduced the viral load 1 day after initiation of treatment compared with oseltamivir [8], the secondary attack rates (Table 3) were similar in both baloxavir and oseltamivir groups. These findings imply that the virological benefit of baloxavir has a smaller effect on household transmission.

In baseline characteristics, the two groups were comparable in influenza vaccination in the same season (Table 4). Therefore, influenza vaccination may be unable to prevent household transmission. A past report showed similar results [15].

According to the multivariate analysis, age 0–6 years was a risk factor for secondary influenza infection (Table 5). This result is similar to that reported previously. Papenburg et al. reported that young children were at the highest risk of influenza-like illness in laboratory-confirmed H1N1 pandemic [2]. Similarly, Viboud et al. reported that the risk of secondary attack was higher in the 0–5-year age group [7]. We noted that it might be difficult for younger children to avoid family contact in their lives, hence the high risk of household influenza infection. On the contrary, baloxavir treatment was not associated with reduced household transmission. Nishimura et al. reported that treatment with zanamivir inhaler reduced the risk of household transmission, unlike oseltamivir [6]. Hirotsu et al. reported that peramivir injection or treatments with zanamivir inhaler lowered secondary infection rate compared to oseltamivir [7]. However, the ability of neuraminidase inhibitor peramivir to reduce the viral load more rapidly than oseltamivir has not been consistently demonstrated in previous studies [16, 17]. Therefore, other factors may be involved in the reduction of household transmission of influenza virus.

Ikematsu et al. reported the postexposure prophylactic efficacy of baloxavir in preventing influenza in household contacts of patients with influenza [18]. However, this study did not indicate the effect of the antiviral drugs administered to index patients on disease transmission. Our findings show that baloxavir is an effective antiviral drug for preventing household transmission of influenza.

Our study has several limitations. First, the results were based on a retrospective review of records and data obtained telephonically. Second, the study was single-centered, with a period of 1 year, limiting the number of patients enrolled and the generalizability of our study findings. Therefore, long-term prospective studies involving multiple institutions with more participants are required. Third, although influenza A was defined using a routine diagnosis kit in this study, details on influenza A subtypes, antiviral drug susceptibility, and viral load were not confirmed. In 2018–2019 in Japan, prevalent influenza subtypes were A/H3 and A/Hapdm09 [19]. The National Institute of Infectious Diseases reported that 8% of influenza A (H3N2) cases were resistant to baloxavir; however, the virus was not resistant to oseltamivir [20]. The reduced susceptibility sometimes causes a rebound in viral titers and prolongation of symptoms [8]. Therefore, further studies are needed to evaluate the impact of drug resistance on the household transmission of influenza.

## Conclusion

The household secondary attack rate of influenza was comparable in patients treated with baloxavir and those treated with oseltamivir. Therefore, baloxavir can be used as an alternative therapy to oseltamivir in reducing household transmission of influenza.

## Data Availability

All data generated or analyzed in this study are included in this published article.

## Abbreviations

RIDTs: Rapid influenza diagnostic tests
CI: Confidence intervals
ROC: Receiver Operating Characteristic
ORs: Odds ratios
IQR: Interquartile range
